# B7-H4 Treatment of T Cells Inhibits ERK, JNK, p38, and AKT Activation

**DOI:** 10.1371/journal.pone.0028232

**Published:** 2012-01-04

**Authors:** Xiaojie Wang, Jianqiang Hao, Daniel L. Metzger, Ziliang Ao, Lieping Chen, Dawei Ou, C. Bruce Verchere, Alice Mui, Garth L. Warnock

**Affiliations:** 1 Department of Surgery, University of British Columbia, Vancouver, Canada; 2 Department of Pediatrics, University of British Columbia, Vancouver, Canada; 3 Department of Pathology and Laboratory Medicine, University of British Columbia, Vancouver, Canada; 4 Department of Immunobiology, Yale University School of Medicine, New Haven, Connecticut, United States of America; Saint Louis University School of Medicine, United States of America

## Abstract

B7-H4 is a newly identified B7 homolog that plays an important role in maintaining T-cell homeostasis by inhibiting T-cell proliferation and lymphokine-secretion. In this study, we investigated the signal transduction pathways inhibited by B7-H4 engagement in mouse T cells. We found that treatment of CD3^+^ T cells with a B7-H4.Ig fusion protein inhibits anti-CD3 elicited T-cell receptor (TCR)/CD28 signaling events, including phosphorylation of the MAP kinases, ERK, p38, and JNK. B7-H4.Ig treatment also inhibited the phosphorylation of AKT kinase and impaired its kinase activity as assessed by the phosphorylation of its endogenous substrate GSK-3. Expression of IL-2 is also reduced by B7-H4. In contrast, the phosphorylation state of the TCR proximal tyrosine kinases ZAP70 and lymphocyte-specific protein tyrosine kinase (LCK) are not affected by B7-H4 ligation. These results indicate that B7-H4 inhibits T-cell proliferation and IL-2 production through interfering with activation of ERK, JNK, and AKT, but not of ZAP70 or LCK.

## Introduction

B7-H4 is an inhibitory member of the B7 family of co-regulatory molecules which is expressed on antigen-presenting cells as well as on non-immune cells and which interacts with an as yet unidentified receptor(s) on activated T cells to inhibit T-cell proliferation and IL-2 production [1]–[4]. The importance of B7-H4 in regulating immune responses *in vivo* has been shown through many studies. Administration of a B7-H4 mAb, that blocks B7-H4 action, to an experimental autoimmune encephalomyelitis (EAE) mouse model promoted T-cell responses and exacerbated disease [2]. Adenoviral-mediated transduction of islets with B7-H4, on the other hand, protected them from rejection when transplanted into allogeneic mice [5]. Studies into the mechanisms by which B7-H4 engagement prevents T-cell proliferation have shown that cells are arrested at the G0/G1 phase of the cell cycle [1]. Addition of exogenous IL-2 can partially reverse B7-H4–induced suppression of T-cell proliferation, suggesting that inhibition of IL-2 production is an important component of B7-H4 action on T cells.

Ligation of the T-cell receptor (TCR) in conjunction with co-stimulatory receptors initiates a cascade of signal transduction events that result in IL-2 production and T-cell clonal expansion and differentiation [6]. The tyrosine kinase LCK is the first signaling molecule to be activated downstream of the TCR [7]. Activated LCK phosphorylates ITAM motifs in the cytoplasmic domain of the TCR gamma, epsilon and zeta chains [8]. ZAP70, another tyrosine kinase, is recruited to the phosphorylated zeta chain and is activated by phosphorylation of LCK. ZAP70 then phosphorylates a number of downstream signaling molecules [9], activating a signaling cascade which includes ERK and JNK kinases, which leads to stimulation of IL-2 transcription [10]. However, these signaling pathways downstream of the TCR network work in conjunction with signaling pathways downstream of the co-stimulatory receptors. One of the key co-stimulatory receptors is CD28, a positive signaling member of the B7 co-regulatory family. CD28 interacts with its cognate ligands (CD80 and 86) on antigen-presenting cells, leading to activation of phosphatidylinositide 3-kinase (PI3K). PI3K catalyzes the production of phosphoinositol-3,4,5-triphosphate (PIP3) which functions to activate PH domain–containing proteins such as the protein kinase AKT. AKT is a master regulator involved in protein synthesis, anti-apoptosis, cell survival/proliferation, and glucose metabolism. Activation of PI3K/AKT pathway is a fundamental requirement for cell-cycle progression and T-cell proliferation.

TCR activation in the absence of CD28 stimulation results in impaired or altered T-cell responses ranging from decreased proliferation/IL-2 production to anergy (non-responsiveness to antigen) or apoptosis. The requirement for co-stimulatory receptor signaling has been used to modulate T-cell responses for therapeutic purpose. For example, much work has been focused into clinical development the CTLA-4 molecule. CTLA-4 is another member of the B7 family, but it functions to inhibit T-cell activation. CTLA-4 is a surface protein that can be expressed on activated T cells and competes with CD28 for binding to CD80/86. CTLA-4 binds to CD80/86 but does not activate PI3K signalling. Soluble versions of CTLA-4 have been used clinically to interfere with T-cell responses in autoimmunity and organ transplantation. Studies of the signaling pathways which are altered in T cells exposed to soluble CTLA-4 have confirmed interference with PI3K-dependent events and also revealed an inhibitory effect on ERK and JNK activation [11].

The signaling pathway(s) by which B7-H4 alters T-cell responses, have not been well characterized. Based on our knowledge of how the other inhibitory B7 family members interfere with T-cell activation, we anticipate that B7-H4–mediated signaling may inhibit MAPK and AKT kinases. In this paper we examine this possibility and whether B7-H4 signaling also alters LCK or ZAP70 activation. Characterization of the molecular mechanism by which B7-H4 functions will provide important information guiding rational use of B7-H4 therapy for cancer, autoimmune diseases and transplantation.

## Results

### B7-H4 engagement inhibits T cell proliferation

**Figure 1:**
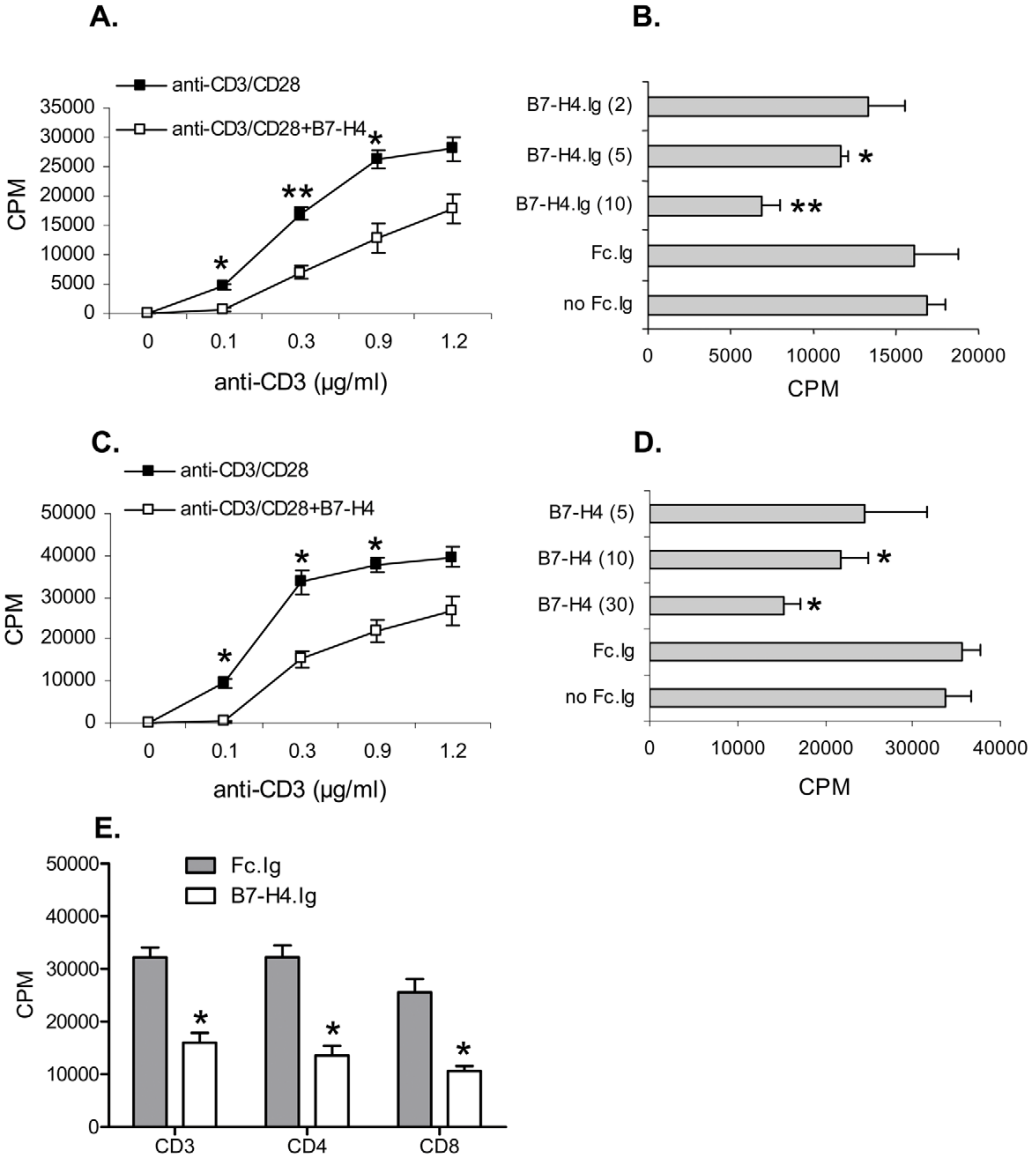
B7-H4 inhibits T-cell proliferation. Naïve (A and B) and pre-activated (C,D, and E) CD3^+^ T cells were stimulated with various concentrations of plate-bound anti-CD3 and soluble anti-CD28 (1 µg/mL) for 72 h. 18 h before harvest, cultures were pulsed with 1 µCi of [3H]-thymidine. B7-H4.Ig or Fc.Ig was added at indicated concentrations (B and D). 10 µg/ml and 30 µg/ml of B7-H4.Ig or Fc.Ig was added for naïve and pre-activated T cells, respectively. Pre-activated CD3^+^, CD4^+^, or CD8^+^ T-cell subsets were stimulated with 0.3 µg/ml of plate-bound anti-CD3 and soluble anti-CD28 (1 µg/ml). Triplicate wells were harvested and counted. Data represent three independent experiments and expressed as means ± SEM. One star (*) indicates p<0.05, two stars (**) indicate p<0.01, and three stars (***) indicate p<0.001.”

We and others have shown previously that administration of a soluble immunoglobulin fusion protein of B7-H4, B7-H4.Ig, inhibited CD4^+^ and CD8^+^ T-cell proliferation upon TCR/CD28 ligation [1]–[4]. However a question that has not been addressed is whether the inhibitory effect of B7-H4.Ig administration affects naïve and pre-activated T cells equally. We thus compared the inhibitory effect of B7-H4.Ig on naïve splenic CD3^+^ T cells versus the same cells which have been stimulated with CD3 for 16 h and rested for 30 h prior to use. Naïve or pre-activated cells were cultured with a range of concentrations of anti-CD3 agonistic antibody, in the presence of a constant concentration of an agonistic CD28 antibody, and with B7-H4.Ig or a control human IgG1 Fc.Ig protein (Fc.Ig). As shown in Figure 1A and 1C, although the calculated EC50 for CD3 was similar in both the naïve and pre-activated cells (∼0.15 µg/mL), maximum thymidine incorporation was different. Thymidine incorporation plateaued at 40×10^3^ cpm for pre-activated cells as compared to 27×10^3^ cpm for the naïve cells. Thus the pre-activated cells proliferate much more vigorously than the naïve cells. However, the ability of B7-H4.Ig to inhibit thymidine incorporation appeared to be similar in both naïve and pre-activated cells. The addition of B7-H4.Ig to the culture reduced thymidine incorporation by almost 50% in the presence of 0.3 µg/mL anti-CD3 in both cell types.

We also tested the sensitivity of naïve vs. pre-activated cells to B7-H4.Ig. In Figure 1B and 1D, the indicated concentration of B7-H4.Ig was added to cultures in the presence of 0.3 µg/mL anti-CD3 and 1 µg/mL anti-CD28. As seen in Figure 1B and 1D, the naïve cells were more sensitive to B7-H4.Ig inhibition. At 10 µg/mL B7-H4.Ig, thymidine incorporation was inhibited by 60% in naïve cells (p = 0.005). In contrast, the same concentration of B7-H4.Ig inhibited thymidine incorporation in pre-activated cells by only 40% (p = 0.012). For the remainder of the studies in this paper, we chose to use pre-activated cells because these cells represent a more stringent system (i.e. cells are more resistant to B7-H4.Ig) with which to examine the mechanism by which B7-H4.Ig interferes with T-cell activation.

In order to determine the sensitivity of inhibition by B7-H4 on different T-cell subsets, CD3^+^, CD4^+^, or CD8^+^ T cells were purified by magnetic beads. As shown in Figure 1E, B7-H4.Ig protein inhibited thymidine incorporation in each subset to a similar degree. The proliferation of total pre-activated CD3^+^, CD4^+^, and CD8^+^ was reduced by 50.3%, 57.9%, and 58.5%, respectively. There were no significant differences in the ability of B7-H4 to inhibit three T-cell subsets (p = 0.6331).

**Figure 2:**
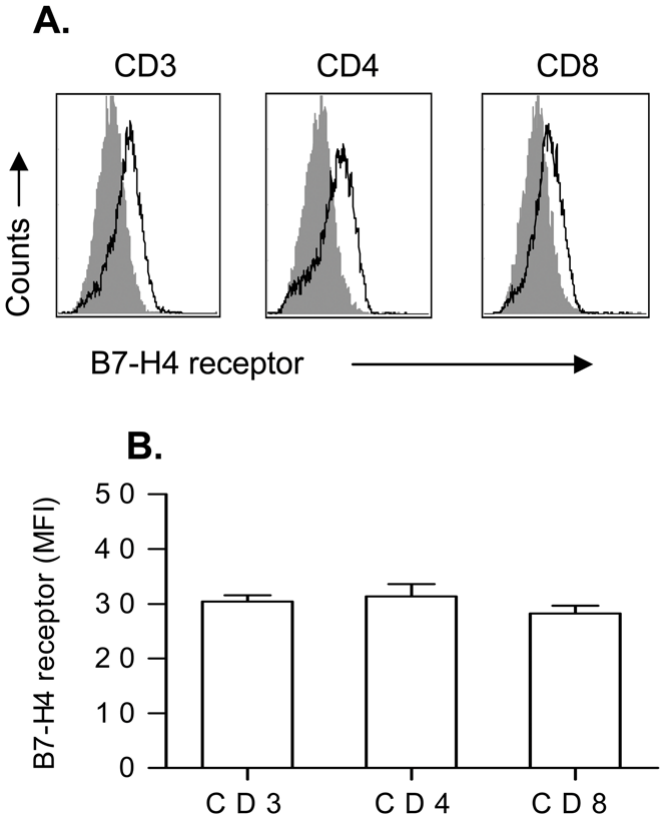
Expression of B7-H4 receptor on activated T-cell subsets. Naïve CD3^+^, CD4^+^, or CD8^+^ T-cell subsets were stimulated with 0.3 µg/ml of plate-bound anti-CD3 and soluble anti-CD28 (1 µg/ml) for 16 h and then rested for 30 h prior to re-activation. Activated CD3^+^, CD4^+^, or CD8^+^ T-cell subsets were stimulated with plate-bound anti-CD3 (5 µg/ml) and soluble anti-CD28 (2 µg/ml) for 4 d. Expression of putative B7-H4 receptor was detected as follows. Cells were stained with a control human IgG1 (filled) or with a B7-H4.hIgG1 (open), followed by goat anti-human IgG-PE. (A). Representative histograms of expression of B7-H4 receptor on CD3^+^, CD4^+^, or CD8^+^ T-cell subsets. (B). Mean fluorescence intensity (MFI) of B7-H4 receptor was plotted. There is no significant difference in the expression of B7-H4 receptor among three different T-cell subsets. Data represent 3 independent experiments and are expressed as means ± SEM.

We also examined the expression of the B7-H4 receptor on pre-activated CD4^+^ vs. CD8^+^ T-cell subsets (Fig. 2). The B7-H4 receptor has not yet been identified, but it can be detected through its binding of B7-H4.g [3]. Briefly, cells were incubated with 10 µg/mL of B7-H4.Ig or control Fc.Ig for 30 min, prior to washing and detection of bound B7-H4.Ig using a PE conjugated goat anti-human Ig Ab. B7-H4 receptor was detectable on pre-activated CD3^+^, CD4^+^, or CD8^+^ T cells starting at 3 days and peaking at 4 to 5 days after stimulation (Fig. 2A). The mean fluorescence intensity (MFI) of B7-H4.Ig staining was similar in all three different subsets (Fig. 2B, p = 0.55), indicating similar expression of the B7-H4 receptor on pre-activated CD3^+^, CD4^+^, or CD8^+^ T cells. The inability to detect B7-H4.Ig binding prior to 3 days likely reflects the limitation of sensitivity of staining method dependent on B7-H4.Ig protein binding followed by detection of the bound B7-H4.Ig using an anti-Ig Ab. Our proliferation data above indicates that cells are inhibited by B7-H4.Ig even at day 0 (naïve cells) after CD3/CD28 activation, at which time B7-H4.Ig protein binding can't be detected using current methods.

**Figure 3:**
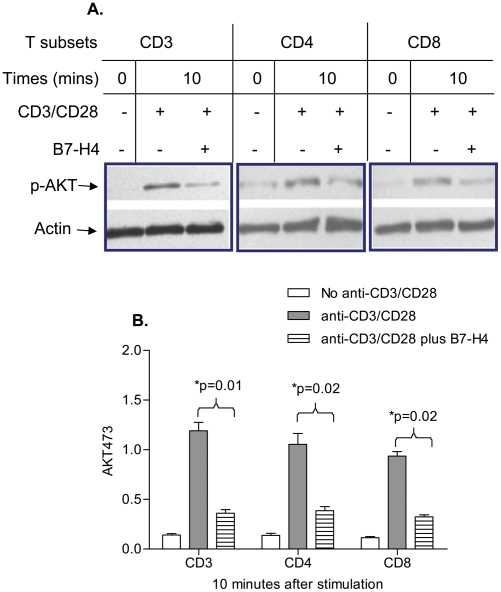
B7-H4 inhibits phosphorylation of AKT on different T cell subsets similarly. Naïve CD3^+^, CD4^+^, or CD8^+^ T-cell subsets were stimulated with 0.3 µg/ml of plate-bound anti-CD3 and soluble anti-CD28 (1 µg/ml) for 16 h and then rested for 30 h prior to re-activation. Activated CD3^+^, CD4^+^, or CD8^+^ T-cell subsets were stimulated with plate-bound anti-CD3 (5 µg/ml) and soluble anti-CD28 (2 µg/ml) for 10 min. Representative western blot of protein extracts from CD3^+^, CD4^+^, or CD8^+^ T-cell subsets were detected by phosphorylated AKT Ser473 (A) and quantitated by using Bio-Rad Quantity One program. The y-axis was normalized for the loading control. B7-H4 treatment significantly inhibited AKT phosphorylation at 10 min to a similar degree on different T-cell subsets (B). Data represent 3 independent experiments and are expressed as means ± SD from 3–4 mice per group.

We also compared the responsiveness of CD3^+^, CD4^+^, or CD8^+^ to B7-H4.Ig inhibition using readout that is more immediate than proliferation assays. CD3^+^, CD4^+^, or CD8^+^ were stimulated with anti-CD3 for 16 h and rested for 30 h prior to use. Cells were then stimulated with 5 µg/mL anti-CD3 and 2 µg/mL anti-CD28 in the presence of 30 µg/mL Fc.Ig or B7-H4.Ig for the indicated length of time. Cell lysates were prepared and subjected to Western blot analysis to determine the levels of CD3/CD28 induced AKT phosphorylation on serine 473 using an AKT-phospho-AKT-ser-473 Ab. AKT is a serine/threonine protein kinase that is essential for multiple cellular processes including the progression of cell cycle and T-cell proliferation and phosphorylation on Ser-473 is a marker of its activation state (16). As shown in Figure 3, anti-CD3/CD28 stimulation could induce AKT-Ser-473 phosphorylation within 10 min. In the presence of B7-H4.Ig, the level of p-AKT-Ser-473 was reduced by ∼70% compared to the Fc.Ig control in all three different T-cell subsets (Fig. 3B).

Our data show that CD3^+^, CD4^+^, or CD8^+^ T cells all express similar levels of the B7-H4.Ig receptor (Fig. 2) and respond to similarly with respect to inhibition of AKT phosphorylation (Fig. 3). We therefore used total CD3^+^ T cells in the following experiments.

### B7-H4 engagement inhibits IL-2 production on T cells

**Figure 4:**
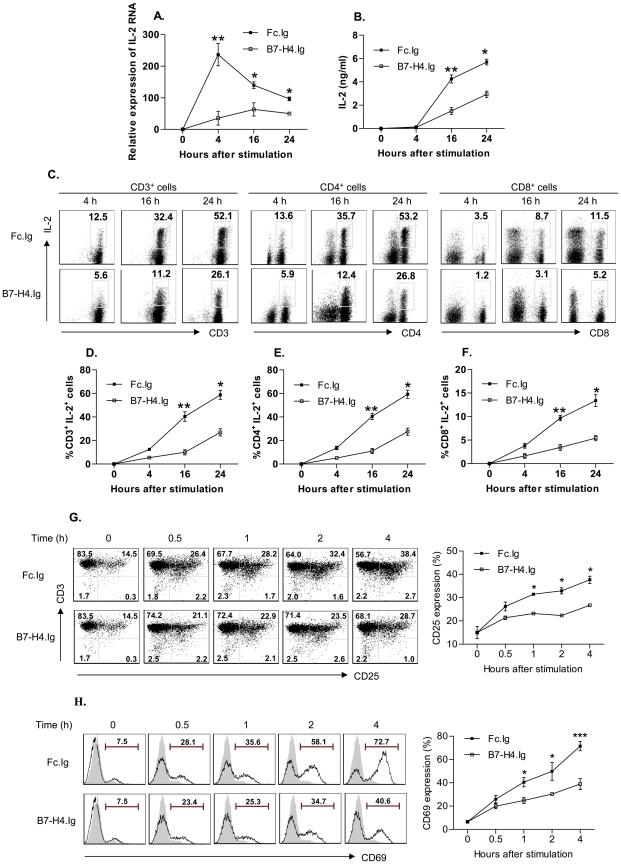
B7-H4 inhibits IL-2, IL-2 receptor α chain (CD25), and CD69 expression. Pre-activated CD3^+^ T cells were stimulated with anti-CD3 (5 µg/mL) and anti-CD28 (2 µg/mL) in the presence of B7-H4.Ig or Fc.Ig. Cells were collected for IL-2 mRNA detection (A) or IL-2 secretion (B), or stained with anti-IL-2 (C, D, E, and F) or anti-CD25 (G) or CD69 (H) at the indicated time points. Relative expression of IL-2 mRNA was detected by real-time PCR (A). Culture supernatant was collected and tested by ELISA (B). Representative dot plot of the expression of IL-2 secretion on CD3^+^, CD4^+^, CD8^+^ population and percentage of double positive IL-2 on three different subsets was shown (C). The average expression level of IL-2 on CD3^+^, CD4^+^, and CD8^+^ was shown in D, E, and F, respectively. Representative dot plot of CD25 expression and average expression level was shown (G). H. Representative histogram of the expression of CD69 was plotted and the average expression level was quantitated using Bio-Rad Quantity One program (H). 20,000 of Living cells were analyzed by flow cytometry. Data represent 3 independent experiments and are expressed as means ± SD. One star (*) indicates p<0.05, two stars (**) indicate p<0.01, and three stars (***) indicate p<0.001.

We next determined the effect of B7-H4.Ig treatment on anti-CD3/CD28–stimulated T-cell production of IL-2. Pre-activated T cells were treated with 5 µg/mL anti-CD3 and 2 µg/mL anti-CD28 in the presence of 30 µg/mL Fc.Ig or B7-H4.Ig for the indicated length of time (Fig. 4A). RNAs were isolated and the level of IL-2 mRNA was quantitated by quantitative PCR. In the anti-CD3/CD28+Fc.Ig cells, IL-2 mRNA levels peaked at 4 h and declined over the course of 24 h (Fig. 4A). The presence of B7-H4.Ig dramatically reduced the amount of IL-2 mRNA detected at 4 h, 16 h, and 24 h (p = 0.001, 0.03, and 0.05). We also collected the cell culture supernatant and measured IL-2 protein levels by ELISA. As shown in Figure 4B, B7-H4.Ig also significantly decreased IL-2 protein detected in the culture supernatant at 16 h and 24 h (p = 0.002 and 0.02, respectively). In order to distinguish the relative contribution of different T cell subsets on IL-2 secretion at single cell level, intracellular staining of IL-2 was performed (Fig. 4C, 4D, 4E, and 4F). In concordance with ELISA data (Fig. 4B), B7-H4 showed the most profound inhibition on three T cell subsets at 16 h after stimulation (inhibition of 75.4%, 72.8%, and 71.5% on CD3^+^, CD4^+^, and CD8^+^, respectively). At 4 h, a similar low level of IL-2 secretion from T cells was observed using ELISA or FACS method (Fig. 4B, 4C, 4D, and 4E).

Activated T cells up-regulate the IL-2 receptor alpha chain CD25 needed for high affinity binding of IL-2. Therefore, we investigated whether the addition of B7-H4.Ig would interfere with expression of CD25. Naïve cells were stimulated and rested as described above. CD25 increased from 6% to 40% within 16 h, but they decreased back to 15% during the 30-h rest period (data not shown). Cells were then treated with anti-CD3/CD28 with Fc.Ig or B7-H4.Ig as before, and the percent of cells expressing CD25 were determined by flow cytometry. As shown in Figure 4G, at 4 h after stimulation, the percent of cells which have upregulated was 37% in Fc.Ig–treated cells vs. 26% in B7-H4.Ig–treated cells (p = 0.03). The relative lower level of inhibition on CD25 (Fig. 4G, 29.7%) compared with on IL-2 (Fig. 4C, 4D, 4E, and 4F, 50–75%) suggested that inhibition on CD25 by B7-H4 might simply reflect the inhibition on T cell activation rather than direct effect on IL-2 receptor. Alternatively, the ability of B7-H4 on inhibition of IL-2 receptor alpha chain is relatively weak compared with its ability to inhibit IL-2.

### B7-H4 engagement inhibits expression of the early activation marker CD69

CD69 is another cell-surface molecule, the expression of which is transiently induced on T cells early during the activation process [12]. We also monitored CD69 expression in the cells used for the CD25 expression studies above. In the pre-activation period, the percent of cells expressing CD69 increased from 7% to 65% at 16 h (data not shown), decreasing almost to basal levels after a 30-h rest (data not shown). Upon re-stimulation with anti-CD3/CD28 (Fig. 4H), the percent of cells expressing CD69 increased to 71% in anti-CD3/CD28+Fc.Ig cells compared with 39% in anti-CD3/CD28+B7-H4.Ig at 4 h (p = 0.0004).

### ERK activity is reduced upon B7-H4 engagement

**Figure 5:**
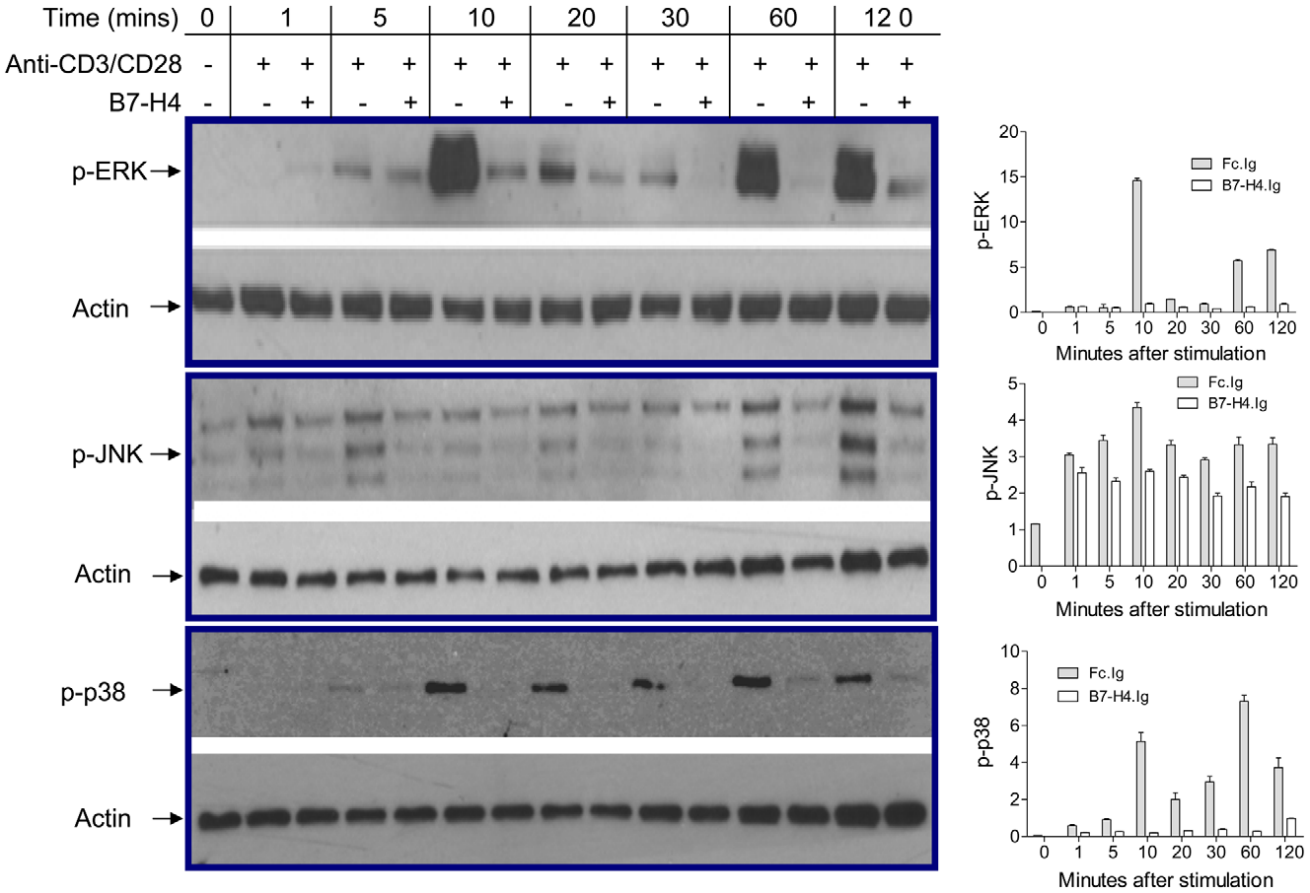
B7-H4 suppresses activation of ERK, JNK, p38, and AKT. Western blot analyses of protein extracts from CD3^+^ T cells stimulated with immobilized anti-CD3 (5 µg/mL) and soluble anti-CD28 (2 µg/mL). Fc.Ig or B7-H4.Ig was added after plate-bound anti-CD3 incubation. Representative western blot for the detection of phosphorylated ERK1/2 Thr202/Tyr204, JNK Thr183/Tyr185, p38 Thr180/Tyr182, AKT Ser473, and GSK-3α/β Ser21/9 as indicated in the graph. p-ERK, JNK, p38, AKT, and GSK-3α/β activity were quantitated by using Bio-Rad Quantity One program. The y-axis was normalized for the loading control. Data represent 3 independent experiments and are expressed as means ± SD form 10–16 mice per group.

One of the key pathways in transcription and expression of IL-2 involves the ERK1/2 kinases [13], [14]. Therefore, we investigated whether or not ERK1/2 activation would be affected by B7-H4 ligation. Cells were pre-activated and rested as before, and then stimulated with anti-CD3/CD28 and Fc.Ig or B7-H4.Ig protein. Cells lysates were prepared and subjected to immunoblot analysis with antibodies to the phosphorylated, active form of ERK [phospho-p44/42 MARK (ERK1/2) (Thr202/Tyr204)]. Phosphorylated ERK1/2 (p-ERK1/2) could be detected as early as 5 min, reaching maximal expression at 10 min, and decreasing to minimal levels at 30 min (Fig. 5). After 60 min, the p-ERK expression rebounded to a high level, likely due to the action of autocrine IL-2. The expression of p-ERK1/2 was reduced with B7-H4.Ig engagement at all time points (Fig. 5). For example, at 10 min, p-ERK1/2 reached peak levels. B7-H4.Ig treatment reduced ERK1/2 phosphorylation to 7% of the level seen in the Fc.Ig treated samples.

### JNK activity is reduced upon B7-H4 engagement

JNK kinase is also involved in T-cell activation and IL-2 production [15], [16]. We therefore also examined the effect of B7-H4.Ig treatment on JNK phosphorylation as a measure of its activation state. The samples from the ERK studies described above were subjected to analyses with antibodies to phospho-SAPK/JNK (Thr183/Tyr185). We found that phosphorylation of JNK could indeed be detected as early as 1 min in response to anti-CD3/CD28 stimulation, reaching a maximum at 10 min, and decreasing to a constant level thereafter (Fig. 5). B7-H4 clearly reduced phosphorylation of JNK at all time points (Fig. 5), with 60% of JNK activity remaining at 10 min, demonstrating that B7-H4 also interferes with JNK activity.

### p38 activity is reduced upon B7-H4 engagement

The effect of B7-H4 on the third MAK kinase p38 was also tested in the same lysates. We found that phosphorylation of p38 could be detected as early as 5 min in response to anti-CD3/CD28 stimulation, reaching a maximum at 10 min, and decreasing to a constant but slight lower level thereafter (Fig. 5). B7-H4 clearly reduced phosphorylation of p38 at all time points (Fig. 5), demonstrating that B7-H4 also interferes with p38 activity.

### AKT activity is reduced upon B7-H4 engagement

As described above, CD28 co-receptor activation of the PI3K/AKT pathway is essential for proliferation, IL-2 production and prevention of development of anergy or tolerance. We probed the lysates prepared above with antibodies to the phosphorylated active form of AKT (phospho-AKT-Ser473). Phosphorylated AKT (p-AKT-Ser473) could be seen as early as 1 min, increasing to a plateau level at 60 min, which remained to 120 min in the presence of anti-CD3/CD28 (Fig. 5). At time points up to and including 60 min, B7-H4.Ig treatment significantly inhibited phosphorylation of p-AKT-Ser473 to ∼30% of the level in the Fc.Ig samples (Fig. 5). Interestingly, inhibition at the 120-min time point was only ∼50%.

We next examined the effect of B7-H4.Ig treatment on the phosphorylation state of GSK-3, an endogenous substrate of AKT kinase, as a separate marker of *in situ* AKT kinase activity [17]. Immunoblots prepared as above were probed with antibodies to phosphorylated GSK-3 (phospho-GSK-3α/β) (Ser21/9). The kinetics of activation of GSK-3 phosphorylation in response to anti-CD3/CD28 was similar to that of AKT-Ser473 phosphorylation, suggesting that p-AKT-Ser473 was a good surrogate marker of kinase activity (Fig. 5). More importantly, B7-H4.Ig treatment inhibited p-GSK-3 in the same way it did p-AKT-Ser473, demonstrating that B7-H4.Ig also inhibited phosphorylation and activation of AKT enzymatic activity.

### B7-H4 does not affect early parameters of TCR triggering

**Figure 6:**
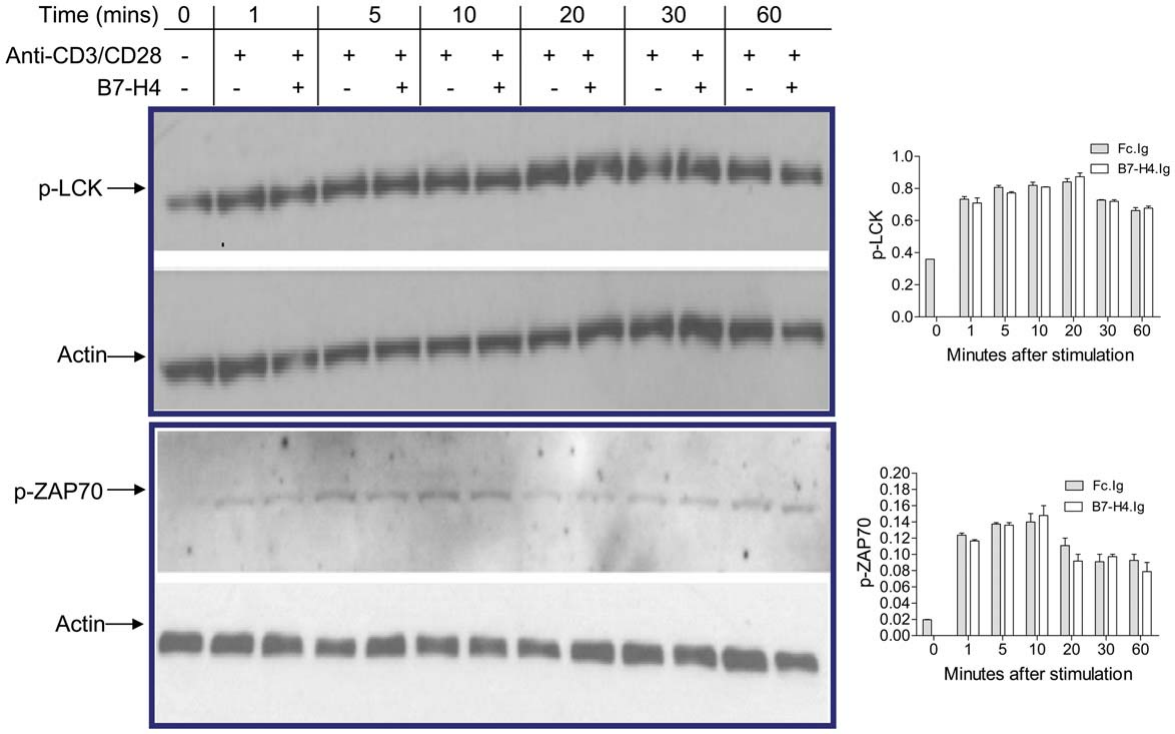
B7-H4 does not affect phosphorylation of LCK or ZAP70. Western blot analyses of protein extracts from CD3^+^ T cells stimulated with immobilized anti-CD3 (5 µg/mL) and soluble anti-CD28 (2 µg/mL) in the presence of control Ig or B7-H4.Ig. Representative western blot for the detection of phosphorylated LCK and Zap70. p-LCK and ZAP70 were quantitated by using Bio-Rad Quantity One program. The y-axis was normalized for the loading control. Data represent 3 independent experiments and are expressed as means ± SD from 10–16 mice per group.

The signaling molecules we examined above are those which CD28 co-receptor signaling regulates (either directly, AKT, GSK-3 or indirectly, ERK, JNK). We next examined the effect of B7-H4.Ig on two signaling molecules which the TCR directly regulates with minimal input from co-receptor action. LCK and ZAP70 tyrosine kinases are directly associated with the TCR complex, and their activation state can be monitored by their phosphorylation state. Phosphorylation of LCK and ZAP70 could be seen as early as 1 min upon anti-CD3/CD28 stimulation, and this was maintained up to 60 min (Fig. 6). B7-H4.Ig treatment did not alter the phosphorylation of either LCK or ZAP-70.

## Discussion

B7-H4 is a new member of the B7 family identified by searching an expressed sequence tagged (EST) database for ESTs with homology to the B7 family [1]–[3], [18]. Investigation into the biological role of B7-H4 revealed that B7-H4 ligation of its as-yet unidentified receptor on T cells inhibits T-cell response and function both *in vitro* and *in vivo* [1]–[5], [19]. Based on these observations, B7-H4.Ig protein is predicted to be able to modify immune responses for therapeutic purpose. Indeed, the addition of B7-H4.Ig protein inhibited the anti-CD3–induced proliferation of activated T cells from patients with type 1 diabetes (T1D), arresting CD4^+^ T cell cycle progression in G0/G1 phase and inducing apoptosis of both activated CD4^+^ and CD8^+^ T cells from these patients. In addition, B7-H4.Ig inhibited the secretion of IFN-γ by PBMC from patients with T1D activated by β cell–associated antigenic (insulin, GAD, and IA-2) peptides, and anti-CD3 antibody. Ectopic expression of B7-H4 in human β cells also protected these cells from cytotoxicity induced by β-cell antigen–specific T-cell clones derived from T1D patients [4]. Local over-expression of B7-H4 in islet allografts by a recombinant adenovirus or insulinoma cell line NIT grafts by gene transduction could similarly inhibit transplant rejection [5], [19]. Tumors have also taken advantage of the immunosuppressive properties of B7-H4. A high level of B7-H4 protein was detected on various cancer cells, including breast, lung, ovary, pancreas, and kidney, suggesting that elevated levels of B7-H4 in the tumor environment may be able to modulate T cells so as to escape immune surveillance [20]–[24]. These studies provide compelling evidence that over-expression of B7-H4 in tumors inhibits T-cell responses through modulation of suppressive APC. The mechanism for this inhibition on T cells by B7-H4 had been proposed to be in part through Treg-stimulated B7-H4 expression on induction of APCs [25]. However, the signaling pathways in T cells altered by B7-H4 ligation have not been well studied.

Production of IL-2 is a hallmark of T-cell activation. The transcription and secretion of IL-2 by activated T cells promotes T cell cycle progression, clonal expansion and effector function. We found that IL-2 mRNA expression increased as early as 30 min in pre-activated T cells upon TCR/CD28 stimulation. The IL-2R is composed of three subunits. Of these three, the CD25 α chain is required for high affinity IL-2 binding, while the β and γ chains are responsible for the transduction of IL-2–generated signals. IL-2R is upregulated by CD3/CD28 ligation through autocrine IL-2–dependent and –independent mechanisms [26]. We found that B7-H4.Ig inhibition of both IL-2 and CD25 expression occurred with similar kinetics but to a different degree, suggesting that IL-2 and CD25 expression may be regulated through autocrine IL-2–independent mechanisms. IL-2 gene transcription occurs in activated T cells through the consequence of various signaling pathways. Both the ERK and JNK kinase pathways have been recognized as important regulators for IL-2 gene transcription. We found in this study that both ERK and JNK activity was stimulated upon anti-CD3/CD28 ligation and reduced in the presence of B7-H4. CTLA-4, another potent negative regulator of the B7 family, has also been reported to inhibit both TCR-induced ERK and JNK activation [11]. It is interesting that B7-H4.Ig inhibited ERK phosphorylation to a greater degree, with only 7% of ERK activity remaining at 10 min (Fig. 5), than JNK phosphorylation. This apparent preferential effect on ERK and JNK and the question of whether B7-H4.Ig treatment leads to inhibition of ERK and JNK kinases directly or via a molecule upstream of these kinases is the subject of future investigation. The inhibition of IL-2 mRNA levels by B7-H4.Ig appears to reduce at 24 h. This may be the result of the action of autocrine IL-2 accumulating in the culture, since the addition of exogenous IL-2 is known to be able to overcome the inhibitory effect of B7-H4 ligation [1]–[3].

CD28 co-receptor signaling is essential for productive T-cell activation, especially of naïve or resting T cells. Blocking CD28 ligation impairs IL-2 production and can lead to T-cell anergy and apoptosis [27], [28]. Inhibition of CD28 co-receptor signaling has been reported to alleviate T cell–dependent diseases [29], [30]. Administration of anti-B7-H4 exacerbated mice EAE disease [2]. We have also shown that B7-H4.Ig addition induces apoptosis of activated CD8^+^ T cells from patients with T1D [4] and that *in vivo* administration to allogeneic islet graft recipient mice is associated with decreased CD8^+^ T-cell levels in the graft and with prolonged survival [5]. Consistent with the well described central role of the PI3K/AKT pathway in CD28 signaling, we found that B7-H4.Ig treatment significantly reduced AKT phosphorylation and kinase activity (as shown by the phosphorylation state of GSK-3). Phosphorylation and activation of AKT is induced directly by CD28 ligation [31]–[33] and also by the action of cytokines such as IL-2 [34]–[36]. Our observation that B7-H4.Ig treatment interferes with AKT activation has a number of important corollaries. First, the observation that exogenously added IL-2 can reverse the effect of B7-H4 action [1] may be due to the fact IL-2 can activate PI3K/AKT via binding to the IL-2 receptor [35], [36] and in this way compensate for the B7-H4.Ig inhibition of CD28-induced AKT activation. Second, the greater sensitivity of naïve vs. pre-activated cells to B7-H4.Ig protein may be related to greater dependence of naïve cells on CD28 signaling [27], [28].

In contrast to the inhibitory effect of B7-H4.Ig treatment on ERK, p38, JNK and AKT activation by anti-CD3/CD28, B7-H4.Ig did not alter the phosphorylation state of LCK or ZAP70 tyrosine kinases. The activation and phosphorylation of these kinases are regulated directly by ligation of TCR itself. LCK is constitutively associated with the cytoplasmic domain of the co-receptor molecules CD4 and CD8 [37] and is the first downstream signaling molecule activated upon TCR ligation [7], [38]. Upon TCR stimulation, LCK (constitutively associated with the CD4/8 chains) autophosphorylates at tyrosine 394 in the activation loop [39], resulting in its activation and the phosphorylation of numerous substrates, include the intracellular domains of the TCR chains. ZAP70 rapidly associates with the phosphorylated CD3, which brings it in proximity to LCK. LCK phosphorylates ZAP70 tyrosine 493 in its activation loop, also resulting in its activation [40]. The phosphorylation state of these tyrosine residues in their activation loops is a strong indicator of their enzymatic activity. We did not observe any effect of B7-H4.Ig on the phosphorylation state of these residues, and it remains to be determined if B7-H4.Ig can alter the ability of these kinases to access certain substrates. We note that the molecules for which we did observe an effect of B7-H4.Ig are either entirely dependent on CD28 signaling (AKT), or they integrate signals from CD28 (ERK and JNK).

In summary, our data show for the first time that B7-H4.Ig interferes with T-cell activation, at least in part through antagonizing signaling pathways downstream of CD28. Future studies will be directed towards characterizing the mechanism by which B7-H4.Ig inhibits activation of AKT, ERK and JNK. From the perspective of CD28 signaling, we will examine whether PI3K activation is impaired and/or AKT phosphatases are activated. From the perspective of B7-H4, its receptor on the T cell needs to be identified and the nature of the signaling pathways downstream of this receptor and/or its physical interaction with CD28 needs to be determined. The current data have provided insight into how B7-H4.Ig protein alters T-cell signaling and function that will be useful to develop therapeutic strategies for B7-H4.Ig. For example, monitoring CD25/69 expression could be a good biomarker for B7-H4.Ig action and B7-H4.Ig might best be used in combination with agents which target the TCR directly, or which target co-receptor signaling in ways different than B7-H4.Ig.

## Materials and Methods

### T-cell stimulation

BALB/c mice were purchased from The Jackson Laboratory and housed in the Jack Bell Research Centre under conventional conditions. All mice were cared for according to the guidelines of the Canadian Council and regulation of the University of British Columbia. CD3^+^, CD4^+^, and CD8^+^ T cells were purified from mouse spleens by using magnetic negative selection (StemCell Inc., Vancouver, BC, Canada). The purity of T-cell subsets was more than 95% by FACS analysis (FACSCanto, Becton Dickinson). CD3^+^, CD4^+^, and CD8^+^ T cells were cultured in tissue culture plates that were pre-coated with anti-CD3ε mAb (145-2C11, BD Bioscience) at indicated concentrations with soluble anti-CD28 (clone 37.51, BD Bioscience). Pre-activated T cells were first stimulated with anti-CD3ε mAb at 0.3 µg/mL. These pre-activated T cells were rested for 30 h before being re-activated with the indicated amounts of plate-bound anti-CD3ε mAb and soluble anti-CD28.

### Purification of B7-H4.Ig fusion protein

B7-H4.Ig was harvested from the supernatant of 293 cells with transient transfection of B7-H4 cDNA in which the extracellular domain was in-frame fused with human Ig G1 Fc fragment. This B7-H4-containing supernatant was purified by Protein A affinity chromatography (Bio-Rad, Mississauga, ON, Canada). The purified B7-H4.Ig was quantitated by SDS-PAGE electrophoresis with bovine serum albumin (BSA) as a standard control and further confirmed by direct ELISA. Control human IgG1.Ig protein was from Cedarlane Laboratories (Cedarlane, Burlington, ON, Canada).

### [^3^H]-thymidine incorporation assay

B7-H4.Ig or control human IgG1 Fc.Ig (Fc.Ig) was incubated in 96-well flat-bottom tissue culture plates that were pre-coated with anti-CD3 antibody for 2 to 4 h at 37°C. Purified T cells (3×10^4^ cells per well) were cultured in RPMI-1640 medium supplemented with 10% FCS, 2 mM glutamine, 100 u/mL penicillin, 100 µg/mL streptomycin and 50 µM 2-mercaptoethanol for 72 h, followed by pulse with addition of 1 µCi [^3^H]-thymidine (PerkinElmer, Montreal, QC, Canada) in the last 18 h. [^3^H]-thymidine incorporation was measured by liquid scintillation counting.

### Cytokine detection

IL-2 mRNA was quantitated by real-time PCR. RNA was extracted with RNeasy mini kit (QIAGEN, Mississauga, ON, Canada). cDNA was synthesized using reverse transcriptase (GIBCO). Quantitative real-time PCR was done in duplicate using 25 ng cDNA with 0.4 µM of each primer in a final reaction volume of 20 µL containing 10 µL of 2× SYBR PCR Master Mix (QIAGEN). PCR parameters were as follows: 50°C for 2 min and 95°C for 10 min, followed by 45 cycles of 95°C for 15 sec, 55°C for 20 sec and 72°C for 30 sec. Relative expression level was calculated as 2^−(CTubiquitin−CTgene)^ (where CT is cycling threshold), with ubiquitin RNA as the endogenous control for normalization. The primer pairs for mouse IL-2 are as follows: forward, CCC ACT TCA AGC TCC ACT TC, and reverse, ATC CTG GGG AGT TTC AGG TT. Mouse GAPDH mRNA was used as an internal control to confirm equivalent loading of the total RNA.

The production of IL-2 protein in the cell supernatants was measured by sandwich ELISA with a pair of anti-IL-2 antibodies (eBioscience, San Diego, CA, USA). Briefly, capture antibodies (clone JES6-IA12) were coated at 2.5 µg/mL on NUNC ELISA plates at 4°C overnight. After three washes, the coated plates were blocked with PBS/3% BSA for 1 h at 37°C. The collected cell supernatants and serial dilutions of recombinant mouse IL-2 standard protein (eBioscience) were added and incubated for 2 h at 37°C. Biotinylated detection antibodies (clone JES6-5H4) were added to capture the IL-2 protein, followed by Streptavidin-HRPO incubation. TMB (Sigma, Oakville, ON, Canada) was used as developing reagent. Absorbance was determined at 405 nm. Triplicate absorbance values of supernatant samples were calculated according to standard recombinant IL-2 concentration in ng/mL.

### Western blot analyses

T cells were lysed in lysis buffer (150 mM sodium chloride,1.0% NP-40, 50 mM Tris, pH 8.0) supplemented with a cocktail of protease and phosphatase inhibitors (Roche, Laval, QC, Canada). Lysates were made in 1× SDS-PAGE buffer and boiled for 2 min prior to separation by SDS-PAGE and electro-transferred to nitrocellulose membranes (Bio-Rad). The amount of protein was determined by Sigma protein assay to ensure equal protein loading in each lane. Antibodies against phospho-ERK (Thr202/Tyr204), phospho-p38 (Thr180/Tyr182), phospho-JNK (Thr183/Tyr185), phospho-AKT (Ser473), phospho-GSK-3α/β (Ser21/9, GSK-3α preferred), and phosphor-ZAP70 (Tyr319) were purchased from Cell Signaling Technology (Danvers, MA, USA). Antibodies to phospho-LCK (Tyr394) were from Santa Cruz Biotechnology. Secondary antibody horseradish peroxidase conjugated goat against rabbit was purchased from R&D (Minneapolis, MN, USA). The blot was developed with Enhanced Chemiluminescence Plus Developer (Pierce, Nepean, ON, Canada) and autographs were quantified using Bio-Rad Quantity One software (Bio-Rad).

### Flow cytometric analysis

2×10^5^ cells were re-suspended in 50 µL ice-cold PBS/1% calf serum buffer and stained with anti-CD3, anti-CD69, anti-CD25, or control rat IgG (eBioscience). Intracellular staining of IL-2 was performed after 4 h stimulation with PMA plus Ionomycin following the standard protocol (BD Biosciences, Mississauga, ON, Canada). 20,000 live gated events were acquired on FACScan, and Devo software was used to analyze relevant populations.

For B7-H4 receptor detection, purified hIgG.Fc or mB7-H4.hIgG.Fc (10 µg/mL) was ligated with T cells for 30′ on ice, followed by incubation with goat anti-human IgG PE for 20′, the expression of B7-H4 receptor was determined by its ability of binding to B7-H4 fusion protein and expressed by mean fluorescence intensity (MFI).

### Statistical analysis

Two-tailed student's t-test or ANOVA was used for comparison between B7-H4.Ig and Fc.Ig treated groups. Data of the secretion of IL-2 protein was analyzed by Prism 4.0 software (San Diego, CA, USA). Differences were considered significant if p<0.05.
